# SleepShifters: The Co-Development of a Preventative Sleep Management Programme for Shift Workers and Their Employers

**DOI:** 10.3390/ijerph22081178

**Published:** 2025-07-25

**Authors:** Amber F. Tout, Nicole K. Y. Tang, Carla T. Toro, Tracey L. Sletten, Shantha M. W. Rajaratnam, Charlotte Kershaw, Caroline Meyer, Talar R. Moukhtarian

**Affiliations:** 1 Warwick Applied Health, Warwick Medical School, University of Warwick, Coventry CV4 7AL, UK; 2 Department of Psychology, University of Warwick, Coventry CV4 7AL, UK; 3 School of Psychological Sciences, Monash University, Clayton, VIC 3800, Australia

**Keywords:** shift work, sleep, early intervention, prevention, workplace, co-production, co-development, digital, health promotion

## Abstract

Shift work can have an adverse impact on sleep and wellbeing, as well as negative consequences for workplace safety and productivity. SleepShifters is a co-developed sleep management programme that aims to equip shift workers and employers with the skills needed to manage sleep from the onset of employment, thus preventing sleep problems and their associated consequences from arising. This paper describes the co-development process and resulting programme protocol of SleepShifters, designed in line with the Medical Research Council’s framework for the development and evaluation of complex interventions. Programme components were co-produced in partnership with stakeholders from four organisations across the United Kingdom, following an iterative, four-stage process based on focus groups and interviews. As well as a handbook containing guidance on shift scheduling, workplace lighting, and controlled rest periods, SleepShifters consists of five key components: (1) an annual sleep awareness event; (2) a digital sleep training induction module for new starters; (3) a monthly-themed sleep awareness campaign; (4) a website, hosting a digital Cognitive Behavioural Therapy for insomnia platform and supportive video case studies from shift-working peers; (5) a sleep scheduling app for employees. Future work will implement and assess the effectiveness of delivering SleepShifters in organisational settings.

## 1. Introduction

In an increasingly globalised society, shift work has become essential to the maintenance of continuous operations, with industrial sectors such as transport, manufacturing, healthcare, and engineering all relying upon shift workers to meet demand and provide 24 h services. While shift patterns vary between organisations, shift workers are often required to work irregular hours and nights, which can disrupt the body clock and cause circadian misalignment [1]. Circadian misalignment can lead to sleep disturbances and insomnia during the sleeping period and excessive sleepiness and fatigue during the waking period [1]. In some cases, these symptoms may progress into Shift Work Disorder, which affects approximately one-quarter of the shift-working population and is accompanied by significant distress and functional impairment [2].

As well as having a negative impact on sleep, shift work-related circadian misalignment can increase the risk of physical and mental health conditions, including cardiovascular disease, cancer, anxiety, and depression [3,4,5,6]. The negative consequences of shift work also extend to the workplace and have been associated with increased risk of accidents and errors, reduced productivity and efficiency, and elevated sickness absence and staff turnover [7,8,9]. As well as compromising workplace health and safety, these impacts result in significant financial losses, and pose an additional burden to the healthcare system and wider economy [10]. Indeed, the health and economic consequences of insufficient sleep are estimated to cost the UK GBP 40 billion each year [11].

Despite the need to address sleep problems in shift workers, standard sleep intervention options are often inappropriate for this population. Sleep medications, for instance, induce sedation rather than naturalistic sleep, and cause significant side effects that harm the individual (e.g., premature mortality and cancer) and hinder workplace output (e.g., reduced concentration and excessive sleepiness) [12]. Sleep hygiene education is also ineffective as a standalone treatment and advises against afternoon caffeine consumption and napping—techniques shift workers often employ to maintain alertness and increase sleep quantity [13]. Additionally, while Cognitive Behavioural Therapy (CBT-I) is the gold standard treatment for insomnia in the general population, it can be inappropriate for shift workers as it involves a consistent sleep–wake schedule and sleep restriction, which may be impossible—and even harmful—to those already experiencing reduced sleep [14]. Irregular schedules and daytime sleeping can also make attending in-person CBT-I sessions difficult, with the scarcity of trained professionals further limiting access [15].

Standard sleep interventions are therefore limited in their applicability to shift workers, one of the most sleep deprived working populations in the world [16]. They are also restricted in terms of their timing (after sleep problems arise), setting (outside of the workplace), and target (individual-level). Indeed, given that organisation-level (e.g., shift schedules, workplace culture) and individual-level factors (e.g., cognitions and behaviours) contribute to sleep problems, early interventions addressing both are likely to be of greater benefit for this population [17,18].

However, while the NHS provides general health recommendations for shift workers employed in healthcare organisations [19], and while standard Working Time Regulations put a limit on the total number of hours worked [20], there are currently no official policies or programmes dedicated to preventing sleep-related issues in the shift-working population in the United Kingdom. Additionally, while new research has adapted sleep hygiene guidelines for shift workers [13], they have not been tested, enforced, or implemented into current standards. Advice on nutrition and exercise also remains generalised.

When it comes to managing sleep and fatigue at the organisation-level, adjusting shift schedules and manipulating workplace lighting has been shown to help minimise the effects of shift work-related circadian misalignment [21]. Other techniques, such as the implementation of planned nap periods and caffeine consumption have also been employed to reduce sleep pressure and manage alertness on-shift (while a comprehensive overview of research-backed recommendations for shift workers is beyond the scope of the current manuscript, we refer readers to the review that was undertaken prior to intervention co-development [21]). Despite the existence of multiple strategies, attempts to combine and integrate these strategies into a preventative, multicomponent programme have remained scarce until more recently and remain subject to limitations [22,23].

While efforts to develop complex interventions for shift workers are now in progress, they remain focused on the reduction of clinical disorders in specific populations (e.g., the reduction of Shift Work Disorder in NHS healthcare staff) [24], as opposed to the prevention of sleep problems in the wider shift working population. Other programmes have not engaged stakeholders as part of a collaborative research partnership during the development phase (e.g., a trial targeting drivers from a public transport company) [25]. Indeed, insights from lived experience groups have been neglected in this research area, meaning the needs, wants, and preferences of shift workers and their employers have been overlooked when it comes to the development of intervention components, their ideal format, and mode(s) of delivery [14,17,18].

Furthermore, shift workers employed in blue-collar industries (e.g., civil-engineering, construction, manufacturing sectors) have been less well represented, despite being at greater risk of health inequalities and disparities [26]. Accordingly, collaborative research solutions that seek to understand and address the challenges faced by this more vulnerable working population are needed and map onto government health priorities [26,27].

Considering the above, this paper describes the co-development of SleepShifters and outlines the resulting programme protocol. SleepShifters is a preventative, organisation-level programme that addresses previous limitations and aims to prevent sleep problems and their associated consequences from arising in the wider shift-working population by equipping shift workers and employers with the skills and knowledge needed to manage sleep from the onset of employment.

## 2. Materials and Methods

Intervention development is described in line with the Medical Research Council’s (MRC) guidance for reporting intervention development studies in health research (GUIDED checklist; see Supplementary File S1) [28].

### 2.1. Design

SleepShifters was co-developed between June 2023 and June 2024 in line with Phase 1 of the MRC’s framework for the development and evaluation of complex interventions [29]. Within this framework, each phase has a common set of core elements that must be considered throughout the research process (see Table 1). As recommended, the INDEX guide to robust intervention development was also employed to guide the development phase [29,30]. A combination of “theory and evidence based” and “partnership” approaches to intervention development were taken, with a greater emphasis on the “partnership” approach; specifically, co-production [29,30].

### 2.2. Procedure

#### 2.2.1. Theory and Evidence-Based Approach

Following complex intervention development guidance [29,30,31], a desk review of published research evidence was undertaken before starting to develop the intervention [21]. This enabled the identification of existing interventions and facilitated an understanding of the current theory and evidence-base. To articulate the programme theory (drawing upon the taxonomy of Behavioural Change Techniques; BCTs) [32], a logic model was also drafted and iteratively refined throughout the intervention development process to provide an overview of intervention components, their associated resources/activities, anticipated mechanisms of change, and intended outcomes.

#### 2.2.2. Partnership Approach: Co-Production

In line with the MRC guidelines [29], complex intervention development should incorporate strong and early engagement with stakeholders. As a result, shift-working organisations were invited to take part in the intervention development process from the onset of this project.

Co-production was conducted in line with guidance from the National Institute for Health Research’s (NIHR) advisory group, INVOLVE [33,34,35]. Collaborative working between the research team and authors of this manuscript (academics with expertise in the areas of sleep, shift work, and intervention design and implementation) and stakeholders (employer representatives with expertise in the operational side of shift work and the implementation of workplace health and wellbeing initiatives; and employees with lived experiences of shift work), allowed for knowledge-based (top-down) and experience-based (bottom-up) input to intervention co-development [36].

The collaborative research process was divided into four iterative stages as follows: Stage 1) Start-up and Planning; Stage 2) Understanding Stakeholder Needs, Wants, and Preferences; Stage 3) Expert Review and Consolidation: Protocol Development; Stage 4) Stakeholder Review and Expert Refinement.

Stage 1) Start-up and Planning

Organisations employing shift workers within the Midlands region of the United Kingdom were identified through existing researcher networks (through previous workplace-based projects the PI was involved in) and job search websites. Given that sleep management programmes targeting healthcare workers and transport companies are being developed elsewhere [24,25], and that addressing health disparities and inequalities in blue-collar workers represents a current public health priority [26,27], civil engineering, construction, and manufacturing sectors were purposively prioritised and approached.

A total of 14 organisations were contacted via email with information about the project and an invitation to meet and find out more. Initially aiming for three, four organisations agreed to take part in the collaborative research process at the end of the meeting and remained engaged from project inception to completion (see Table 2 for organisation characteristics).

Collectively, partner organisations spanned civil engineering/construction (*n* = 3), and manufacturing industries (*n* = 1). Civil engineering/construction sector organisations overlapped in terms of the type of work and services they provided; namely, the construction, maintenance, and repair of national infrastructure (i.e., the railway).

While organisations were initially recruited from the Midlands area, due to the nature of their work, civil engineering/construction sector organisations had project sites spanning the United Kingdom. As a result, co-production activities were conducted across the Midlands, the North, and the Southeast of England in line with organisation preferences. A key contact from each organisation supported the recruitment of employees and liaised with the research team to find suitable dates, times, and meeting facilities for co-production activities. To take part, employees were required to be over eighteen years of age. No limits were put on years of shift work experience to obtain lived experience insights across career stages.

Stage 2) Understanding Stakeholder Needs, Wants, and Preferences

Co-production activities were developed in line with the following aims (adapted from [30,31,32]):

(1) To understand the impact(s) of shift work and identify areas of priority.

Employees were asked how shift work had impacted their sleep and/or other areas of their lives (e.g., health, wellbeing, mood, social functioning, behaviours, etc.). Employers were asked how they thought shift work might impact employee sleep, and whether they were aware of any resulting impact(s) on organisation-level outcomes (e.g., health and safety, productivity, accidents, etc.).

(2) To identify potential ways to address priority areas.

To help generate ideas, employees and employers were asked about their current sleep management practices (individual- and/or organisation-level). They were also presented with a series of research-backed intervention suggestions identified by the desk review [21] to explore their potential suitability for a new sleep management programme (e.g., adjusting shift schedules, controlled light exposure, sleep hygiene education, planned napping, caffeine consumption, CBT-I, and mind–body interventions. Stakeholders were further asked to suggest any additional elements that could be incorporated into the new programme.

(3) To identify potential facilitators and/or modes of intervention delivery.

After identifying potential intervention elements that could be included in the new programme, stakeholders were asked to identify potential facilitators and/or modes of delivery that could aid the implementation of these elements within a real-world setting.

(4) To identify potential barriers and facilitators to implementation and engagement—what should a good sleep management programme look like?

As no protocol had been developed at this stage, employees and employers were pre-emptively asked what a good sleep management programme should (or should *not*) look like in terms of format and delivery. Stakeholders were asked to highlight any factors that might encourage or discourage themselves, their colleagues, and/or their organisations from engaging in and/or implementing such a programme, and how these barriers could be overcome.

##### Employee Co-Production Workshops

To gather employee perspectives on the above, five 2 h co-production workshops were conducted across the four organisations, resulting in insights from a total of 26 individuals with lived experiences of shift work (Org A, *n* = 12; Org B, *n* = 6; Org C, *n* = 4; Org D, *n* = 4). Two workshops were held with Org A to ensure both night shift patterns could take part in the co-production process, thus helping to avoid issues of shift rivalry that had been mentioned during the recruitment process. Workshops were hosted at each organisation’s site of choice and were facilitated by two researchers with expertise in sleep research and intervention development (A.F.T. and T.R.M.).

To avoid power imbalances, establish trusting relationships, and provide a safe space for open discussion to take place [33,34,35], employers did not attend employee co-production activities. All sessions started with an introductory icebreaker activity, and the value of employees as lived experience experts was reiterated. Refreshments were made available throughout the sessions, and break times were built-in to minimise fatigue.

To ensure aims were met within the available timeframe, workshop content, materials, and discussion points were standardised across sessions and facilitated by PowerPoint presentations; nevertheless, a fluid approach was adopted, thus providing the flexibility to adapt discussion topics and timings as needed [33]. Multiple activities, resources, and methods of participation were also incorporated into the workshops to provide variation in activity and accommodate individual differences in preferred contribution style (i.e., discussion prompts to promote verbal, group-based conversation; Post-it notes and pens to facilitate group-based brainstorming; and handouts to support individual anonymous written input). Workshops were also audio-recorded via OBS studio to support future consolidation of ideas.

##### Employer Representative Interviews

To gather employer perspectives, eight one-to-one semi-structured interviews (~1 h) were conducted with employers from each of the four organisations, including operations managers (*n* = 2), general managers (*n* = 1), fatigue managers (*n* = 1), and health and wellbeing leads (*n*= 4). The semi-structured interview schedule developed for this study is available in Supplementary File S2. Employer interviews were conducted via Microsoft Teams and audio-recorded via OBS Studio to support future consolidation of ideas.

Stage 3) Expert Review and Consolidation: Protocol Development

After the first round of co-production activities had been conducted, the research team reviewed and consolidated stakeholder input to identify emerging themes in line with each of the four aims. Areas of priority were mapped onto potential intervention components and considered in line with delivery preferences. Potential barriers and facilitators to intervention engagement and/or implementation were also taken into account. Expert review and consolidation of stakeholder inputs facilitated the development of the intervention protocol, including intervention components and associated format and delivery ideas.

Stage 4) Stakeholder Review and Expert Consolidation

To gather thoughts and feedback on the intervention protocol, a second round of co-production activities were conducted. Within these sessions, stakeholders were given an overview of suggested intervention components and asked to provide their thoughts and feedback through a series of discussion prompts and questions relating to component content, format, and delivery. Expert review of consolidated stakeholder input facilitated further refinement of the intervention component content, delivery, and format.

##### Employee Review Workshops

Employees were invited to attend an in-person review workshop, hosted at their respective organisation’s site and facilitated by two researchers (A.F.T. and T.R.M.). Five employee review workshops were conducted, resulting in insights from a total of 23 individuals with lived experiences of shift work, eight of whom had previously attended a co-production workshop in Stage 2 (Org A, *n* = 8; Org B, *n* = 4; Org C, *n* = 3; Org D, *n* = 8).

##### Employer Focus Group

Employer representatives were invited to a joint in-person focus group at the hosting university. A total of eight representatives attended the session, seven of whom had previously taken part in an interview during Stage 2. The focus group was facilitated by two researchers (A.F.T. and T.R.M.) and supported by a live scribe, who visually consolidated refinements to the intervention protocol in real-time.

### 2.3. Ethical Considerations

As employees and employers were contributing to public and patient involvement in research (PPI), ethical approval was not required in line with NIHR guidance [35,36,37]. Accordingly, personally identifying information (i.e., data on age, gender, ethnicity) was not collected. Indeed, stakeholder input was derived from PPI activity mentioned above, and is therefore presented in illustrative figures and tables (as opposed to formal surveys and interviews with associated data tables and codebooks). Nevertheless, standard ethical procedures were followed, and all individuals were required to read a participant information sheet and provide their informed consent before taking part. The researchers also provided a verbal description of the project and gave stakeholders the opportunity to ask questions prior to the commencement of all co-production activities. Individuals were reminded of their right to withdraw at any point, without giving a reason.

Workshops and interviews were audio-recorded to facilitate notetaking, allowing the researchers to actively listen and respond to employees and employers during discussions. Stakeholders were informed that the audio-recordings would only be accessible to the research team and would be stored on secure university servers in password-protected files. Stakeholders were also assured that no personal information would be collected, and that their contributions would not be directly quoted, shared with their organisation, or linked back to them in any way. Audio-recordings were deleted at the end of the project. All employees who attended a co-production workshop were compensated GBP 50 for their time.

## 3. Results

### 3.1. Understanding Stakeholder Needs, Wants, and Preferences: Input from Employee Co-Production Workshops and Employer Representative Interviews

**Figure 1:**
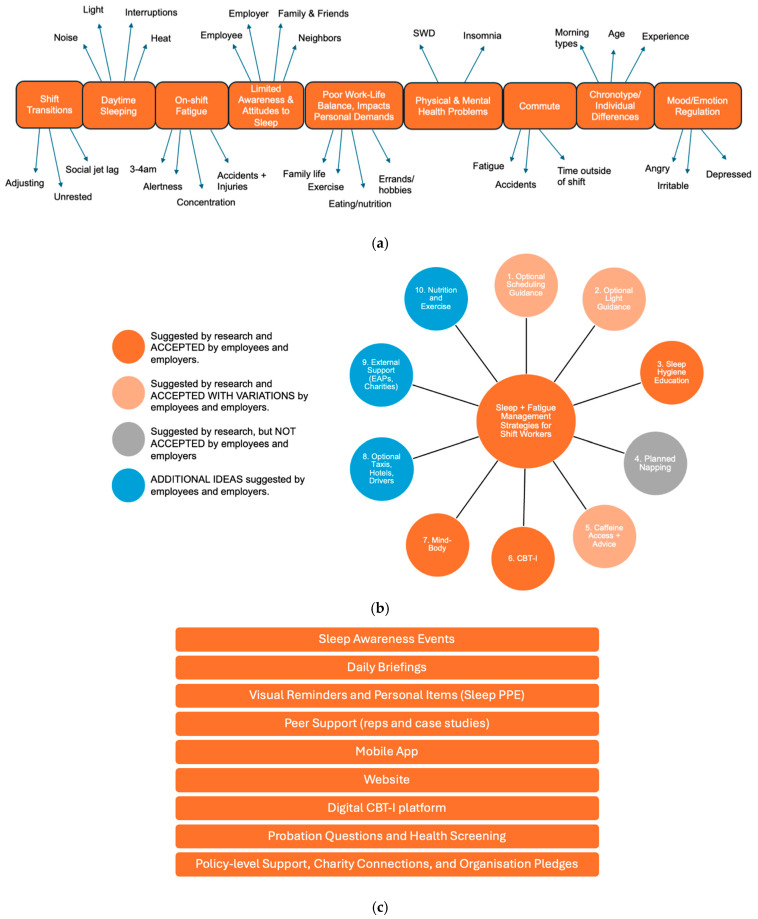

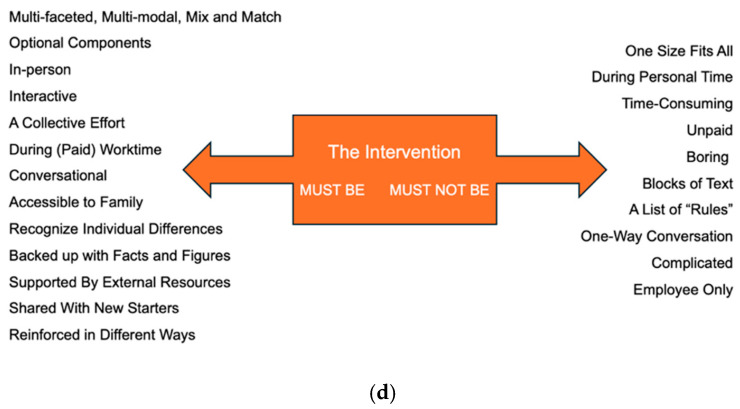
(**a**) Consolidated stakeholder input in relation to aim 1 of Stage 2: (1) Understanding the impact(s) of shift work and identifying areas of priority. Each orange box indicates an issue that employees reported experiencing as a result of working shifts, and therefore represents a priority area for intervention (e.g., issues with “daytime sleeping”). The words around each box provide more context as to how that particular issue manifests for shift workers (e.g., issues with daytime sleeping occur due to “noise”, “light”, “heat”, and “interruptions”). Note: SWD = Shift Work Disorder. (**b**) Consolidated stakeholder input in relation to aim 2 of Stage 2: (2) Identifying potential ways to address priority areas. Note: CBT-I = Cognitive Behavioural Therapy for Insomnia; EAP = employee assistance programme. (**c**) Consolidated stakeholder input in relation to aim 3 of Stage 2: (3) Identifying potential facilitators/modes of intervention delivery. Note: PPE = personal protective equipment; CBT-I = Cognitive Behavioural Therapy for Insomnia. (**d**) Consolidated stakeholder input in relation to aim 4 of Stage 2: (4) Identifying potential barriers and facilitators to implementation and engagement—what should a good sleep management programme look like?

A graphical representation of consolidated stakeholder input, mapped on to each of the four aims outlined in Stage 2 is provided below (Figure 1a–d).

(1) To understand the impact(s) of shift work and identify areas of priority.

In line with the research literature, employees and employers recognised the impacts of shift work on sleep, health, and wellbeing-related outcomes and identified a number of priority areas for intervention (see Figure 1a).

(2) To identify potential ways to address priority areas.

When it came to addressing priority areas, stakeholders felt that research-backed intervention methods such as adjusting shift schedules, controlled light exposure, planned napping, and caffeine consumption would be better delivered as guidance only (as opposed to core components of an intervention) to allow organisations and individuals the flexibility to implement actions in line with their suitability and/or preferences. For instance, while stakeholders from both the civil engineering and manufacturing sector shared the same financial and logistical reservations about reducing shift duration—namely, that reducing shift length would require putting on additional shift rotations, employing more staff, and losing out on the pay that long hours provided—those from the civil engineering sector raised an additional barrier due to the often reactive and emergency-based nature of their work (e.g., on the railway), where work would have to continue until the emergency was resolved. In contrast, sleep hygiene education, CBT-I, and mind–body interventions were well-received (see Supplementary File S3 for an overview of stakeholder reservations).

Indeed, employers questioned why sleep education was not already incorporated into their health and safety training inductions, while employees expressed an interest in learning more about the importance and function(s) of sleep. Providing individuals with access to a therapeutic platform (CBT-I adapted for shift workers) was also deemed to be an appropriate intervention element given the likelihood of serious sleep complaints in this working population. Stakeholders were also receptive to mind–body interventions (i.e., mindfulness, exercise, relaxation techniques).

Employees and employers also identified several additional intervention elements that could be incorporated into the programme based on current organisational practices, including the use of employee assistance programmes, charity connections, and advice on nutrition and exercise (see Figure 1b). Indeed, despite being focused on sleep, stakeholders felt that an intervention for shift workers should include advice on nutrition and exercise, given the impact that shift work can have on appetite and the (in)ability to meet personal demands (i.e., exercising, cooking). Civil engineering firms also considered arranging taxis or hotels for employees to reduce fatigue from long commutes, but employees preferred commuting home, especially if duration of hotel stays were unpaid. Taxis were also considered to be unreliable, with minibuses or designated drivers favoured by employees, although employers were hesitant to allocate drivers and considered pick-up points to be too dispersed for this to be feasible.

(3) To identify potential facilitators/modes of intervention delivery

Stakeholders identified a variety of facilitators/modes that could be used to deliver intervention elements in a real-world setting (see Figure 1c), with employees expressing a preference for in-person delivery methods (e.g., sleep awareness events, educational content and reminders during daily briefings, peer support). Employer representatives suggested that policy-level support, charity connections, and organisational pledges and/or accreditations could also help them to remain accountable and committed to the delivery of new initiatives; however, employees felt accreditations were tokenistic and rarely led to observable benefits.

(4) To identify potential barriers and facilitators to implementation and engagement—what should a good sleep management programme look like?

Employers and employees were very clear on what a good sleep management programme should (or should *not*) look like to facilitate implementation and maximise engagement (see Figure 1d). Stakeholders recognised that approaches to intervention were not a case of ‘one-size-fits-all’ and emphasised the importance of a multicomponent approach that provided tailorable options for organisations and individuals. Indeed, all stakeholders recognised that employee sleep was a collective responsibility, thus requiring support options at the level of the workplace and the individual.

### 3.2. Expert Review and Consolidation: Protocol Development

Following co-production activities, the research team reviewed and consolidated stakeholder inputs, facilitating the articulation of intervention component ideas and associated format and delivery suggestions. Areas of priority (e.g., limited awareness and attitudes to sleep) were mapped onto potential intervention elements (e.g., sleep awareness events and campaigns) and considered in line with delivery preferences (e.g., in-person, during paid work time). To gather stakeholder feedback and facilitate protocol refinement, the researcher team devised a series of discussion prompts and questions relating to the suggested intervention component content, format, and delivery (see Supplementary File S4).

### 3.3. Stakeholder Review and Expert Refinement

**Figure 2:**
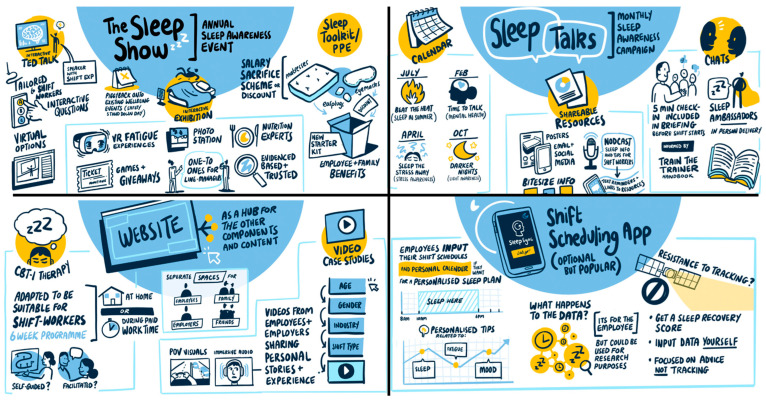
Illustrative visual summary of employer representative feedback and refinement of key programme components. (**Upper Left**): The Sleep Show. (**Upper Right**): Sleep Talks Campaign. (**Bottom Left**): Website. (**Bottom Right**): Shift Scheduling App.

**Figure 3:**
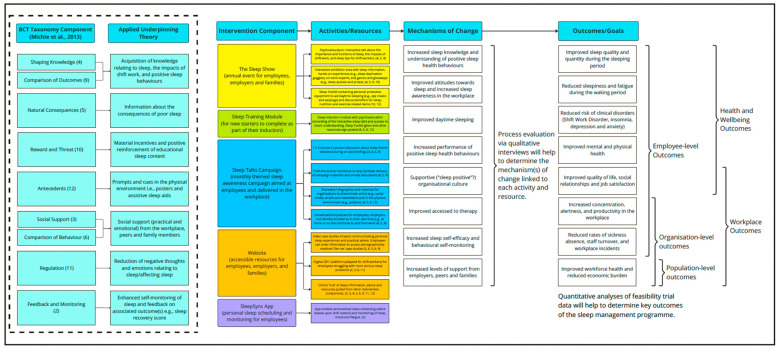
Working logic model for SleepShifters at the end of the co-development phase. BCT = Behavioural Change Technique [32]. Numbers in brackets within figure indicate associated BCT number.

Following stakeholder review and expert consolidation, intervention components, their associated format, and mode(s) of delivery were further refined. Questions posed to stakeholders and a consolidated overview of stakeholder feedback and suggested refinements are provided in Supplementary File S4. The employer focus group was also supported by a live scribe, who created a visual summary of feedback and suggestions in real-time (see Figure 2). A working logic model summarising the refined SleepShifters intervention components, their associated resources/activities, anticipated mechanisms of change, and intended outcomes at the development phase is also provided in Figure 3.

In accordance with MRC intervention development reporting guidance [28], the refined intervention protocol is outlined below in line with the TIDieR checklist (see Supplementary File S5) [38]. Further collaborative research activity with organisational stakeholders will be carried out to co-develop intervention component content and materials (e.g., text, language, look and feel, etc.). Further description of the final intervention protocol will be detailed within a future feasibility assessment paper.

#### 3.3.1. SleepShifters

The co-developed SleepShifters programme consists of five key components:

(1) The Sleep Show:

An annual sleep awareness event for employees, employers, and their families. The Sleep Show will consist of an educational talk and interactive exhibition, featuring games (e.g., sleep quizzes), hands-on experiences (e.g., sleep deprivation goggles), and information stands staffed by sleep experts and clinicians, nutritionists, charities, and sponsors. Attendees will receive a free “Sleep Toolkit” containing personal protective equipment to help facilitate daytime sleeping (e.g., earplugs and eye masks). The toolkit will also contain informative leaflets (e.g., good sleep hygiene practices for shift workers, healthy recipe cards, etc.,), discounts on sleep, diet, and exercise-related items (e.g., mattresses, meal subscription boxes, gym classes), and a reminder of the available sleep resources employees have access to (e.g., QR code to the SleepShifters website).

Organisations will be asked to find a suitable date and hosting venue for the event, which will be facilitated by an external team of sleep experts, researchers, and clinicians. Organisations may wish to ‘piggy-back’ The Sleep Show onto other annual health and safety events and/or training days, where employee attendance is mandatory. Educational sleep content will be tailored to shift workers, with an emphasis on practical, research-backed tips, and consensus guidelines on healthy sleep tips for shift workers [13].

(2) Sleep Smart Induction:

A digital sleep education module for new starters to complete as part of their online health and safety induction training. The interactive module will cover educational sleep, health, and wellbeing content, including highlights from The Sleep Show. As well as receiving a Sleep Toolkit, new starters will also be asked about their sleep as part of their probationary review and line managers will signpost appropriate resources based on individual needs.

(3) Sleep Talks Campaign—We Need to Talk About Sleep:

A monthly themed sleep awareness campaign delivered in the workplace throughout the year. Each month’s theme will be linked to a seasonal event or topic (e.g., how to sleep well in the heat of the summer months). Organisations will be given a train-the-trainer handbook to guide campaign delivery, which will involve a variety of formats to promote exposure and engagement with educational content, including:

Physical Methods: Posters in the physical work environment and five-minute “Sleep Talk” sessions delivered by line managers and/or wellbeing reps during morning briefings. Sessions will involve bite-sized educational content on the sleep topic as well as practical tips and discussion prompts to encourage employees to have open conversations about sleep and associated difficulties in the workplace.

Digital Methods: Shareable infographics for social media channels; educational content to embed into current employee email/newsletter circulations; and podcast episodes. Employees will be reminded of resources and remain free to choose their preferred modes of engagement.

(4) SleepShifters Hub:

An accompanying website, hosting sleep information, tips, and resources will be made accessible to employees, employers, and their families. The website will also host additional sleep support options, including:

Digital CBT-I Platform: A self-guided digital CBT-I platform for individuals requiring a therapeutic level of sleep support (i.e., for insomnia). The platform will employ core principles of CBT-I adapted for shift workers [39]. The online platform will draw upon a pre-existing framework developed by members of the research team (T.R.M., N.K.Y.T.), which has been shown to have significant benefits in other working populations [40,41,42]. Employers will signpost employees to the platform and allow them to engage in self-guided content during worktime. Employees will also be given the option to engage with CBT-I during their own time. A supportive chat function will be monitored by a team of sleep experts, researchers, and/or clinicians to answer questions.

Video Case Studies: For employees seeking practical tips and/or a less formal form of support, video case studies of shift working peers will also be available. Video case studies will contain footage of real shift workers discussing their experiences of shift work-related sleep problems, advice, and practical solutions. To help tailor content, employees will be given the option to access video case studies of peers ‘like them’ by entering their demographic information (e.g., age, gender, race, ethnicity, shift type, years of experience, etc.).

(5) *SleepSync:*

An optional shift scheduling app (currently for research use only) developed by members of the research team (T.L.S., and S.M.W.R.) [42,43,44]. Employees will have the option to download the *SleepSync* app, where they will be able to input their shift rosters and personal commitments to receive tailored sleep tips and advice, such as when to sleep and how long to sleep for. The app also allows employees to self-monitor their sleep quality and quantity, fatigue, and mood, and provides an overall ‘recovery score’. Employers will not have access to employee app data.

#### 3.3.2. Onboarding and Support with Implementation

Organisations will be invited to attend an introductory call, where details of the SleepShifters programme will be provided. Those who wish to proceed with programme implementation will be asked to identify an employer representative(s) who will be responsible for overseeing the programme delivery within their organisation. The representative(s) will then be invited to a training day, where further instruction on the delivery of each intervention component will be provided.

During this session, attendees will be helped to tailor and/or modify aspects of the intervention to suit their organisation’s needs. An intervention handbook will also be provided, and an additional progress check-in with the programme developers will be arranged for later in the year. As well as providing a detailed overview and instructions on how to implement each of the components described above, the handbook will contain guidance on the optional implementation of healthy shift scheduling, workplace lighting, and controlled rest periods.

## 4. Discussion

This paper has described the co-development of SleepShifters, a preventative, organisation-level sleep management programme for shift workers and their employers. Programme development was conducted in accordance with the MRC’s guide to the development of complex interventions [29,30,31]). The SleepShifters programme addresses the limitations of standard sleep interventions and seeks to equip shift workers and their employers with the skills and knowledge to manage sleep from the onset of employment. Indeed, SleepShifters is an early intervention that acts at both universal (workplace) and indicative (individual) levels to prevent sleep problems and their associated consequences from arising [44].

As well as addressing the limitations of standard sleep interventions, this newly co-developed programme also furthers recent efforts to develop complex interventions for shift workers elsewhere [24,25]. Firstly, rather than targeting specific populations (e.g., healthcare workers, bus drivers), SleepShifters can be applied to most shift-working organisations, thus increasing its future scalability and impact. While the programme was purposively co-designed with underrepresented groups in this research area (i.e., civil engineering, construction and manufacturing sector employees and employers), it can be more widely applied and tailored to a variety of shift-working organisations and environments. This inclusive approach helps to provide solutions for a larger variety of shift-working populations, including these groups who may be at greater risk of health inequalities and disparities [26]. Indeed, stakeholder engagement throughout the development phase has helped to ensure that each programme component and its associated format and mode(s) of delivery are in line with the needs of lived experience groups.

Secondly, as opposed to focusing exclusively on individuals already suffering from sleep disorders, the SleepShifters programme is an early intervention that predominantly operates at the universal level. Indeed, ensuring all employees within an organisation—including new starters and current staff—are exposed to intervention resources from the onset of employment increases the likelihood that sleep problems and their associated consequences will be prevented. Nonetheless, given that some shift workers may already be suffering with clinical sleep problems prior to programme implementation, SleepShifters also provides indicative-level options for those who may require—or may be identified as requiring—additional support for their symptoms (i.e., digital CBT-I platform). In addition to evidence that interventions for insomnia can be successfully implemented in workplace settings [41,42,43,44,45], employees felt that organisations should provide access to therapeutic support options by default given the risk to sleep health that shift work poses.

Future work will also be required to determine whether the SleepShifters programme can be successfully implemented within a real-world organisation setting through the exploration of recruitment, retention, and acceptability rates. A future pilot trial employing a mixed-methods approach will be required to track changes in individual- (e.g., sleep, physical health, quality of life) and organisational-level (e.g., sickness absence, workplace accidents/incidents, productivity) outcomes deemed to be important by stakeholders, determine the programme’s cost-effectiveness, understand mechanisms of impact, and gain insights into user experience. Overall, if the programme is found to be acceptable and effective, efforts will be made to roll it out on a larger scale. Ultimately, the goal is to develop a scalable and sustainable programme that can be incorporated into existing health and safety standards of shift working and achieve a positive societal and public health impact.

## 5. Conclusions

SleepShifters is a comprehensive sleep management programme that seeks to address the unique challenges faced by shift workers and improve outcomes for individuals, organisations, and wider society. As well as providing a form of early intervention, this co-developed programme offers a series of tailored components, ranging from educational sleep events to digital tools. Future work will assess the feasibility of implementing SleepShifters within organisational setting to determine its scalability and sustainability for integration into future shift-working standards and practice.

## Figures and Tables

**Table 1:** Mapping core elements of MRC Framework [29] to SleepShifters.

Core Element	Considerations at the Development Stage
Consider Context	Consider the context of employment for shift workers based on industry type and job role, and how this could impact intervention acceptability. Ensure programme content can be modified and tailored to meet the needs of each organisation and have examples relatable to employees across settings.
Develop, Refine, and (Re)test Programme Theory	Through discussion with stakeholders and research team experts, establish a theory of change for the programme and illustrate it via a logic model. Continue to refine the theory of change in subsequent phases through process evaluation with stakeholders.
Engaging Stakeholders	Engage shift workers and employer representatives throughout the development phase via interviews, co-production workshops, and focus groups. Use an iterative process to collaboratively identify and co-develop intervention components, their ideal format, and mode(s) of delivery.
Identify Key Uncertainties	Identify key uncertainties relating to intervention components, their associated format, and mode(s) of delivery through discussion with stakeholders and research team experts (e.g., appropriateness of language, intervention delivery costs, impact of personal demands on sleep schedules and opportunity).
Refine Intervention	Co-develop and refine the intervention protocol in line with stakeholder input and research team experts at all stages of development and beyond (i.e., Phase 2, feasibility trials).
Economic Considerations	Explore economic costs associated with intervention delivery through employer interviews and focus groups (e.g., web hosting costs, staff training, time off shifts to participate in programme elements, etc.). Assess cost-effectiveness in subsequent phases.

**Table 2:** Characteristics of organisational partners.

Organisation	Industrial Sector	Size *	Area of Operation	Location of Co-Production	Shift Pattern	Shift Duration
Org A	Manufacturing	Large	International	Midlands, England	4 on, 4 off (individuals work only nights or only days)	12 h
Org B	Civil engineering/ Construction	Large	United Kingdom, Ireland, and North America	Southeast England	Irregular—mixture of days and nights, up to 14 consecutive shifts	Up to 14 h door-to-door
Org C	Civil engineering/ construction/ rail services	Large	United Kingdom	Midlands, England	Irregular—mixture of days and nights, up to 14 consecutive shifts	Up to 14 h door-to-door
Org D	Civil engineering/construction/	Large	United Kingdom	North England	Irregular—mixture of days and nights, nights mostly on weekends	Up to 12 h door-to-door

* Large organisation = 1001 to 5000 employees.

## Data Availability

Data sharing is not appliable to this article. The original contributions presented in this study are included in the article and Supplementary Materials. Further enquiries can be directed to the corresponding authors.

## Supplementary Materials

The following supporting information can be downloaded at: https://www.mdpi.com/article/10.3390/ijerph22081178/s1, Supplementary File S1: GUIDED Checklist; Supplementary File S2: Employer Interview Schedule; Supplementary File S3: Stakeholder Reservations; Supplementary File S4: Questions Posed to Stakeholders at the Refinement Stage and Associated Feedback; Supplementary File S5: TIDieR Checklist.
