# Supplementary material for: SleepShifters: The Co-Development of a Preventative Sleep Management Programme for Shift Workers and Their Employers
Source: Int J Environ Res Public Health. 2025 Jul 25;22(8):1178. doi: 10.3390/ijerph22081178 (PMC12386607; doi:10.3390/ijerph22081178)
Supplement: Supplementary file 1 [file ijerph-22-01178-s001.zip › Supplementary File S2 – Employer Interview Schedule.pdf]

Supplementary File S2: Employer Representative – Informal, Semi-Structured Interview Schedule

Stage 2) Understanding Stakeholder Needs, Wants, and Preferences

1) To understand the impacts of shift work and identify areas of priority

The impact of shift work on employees as individuals

- How do you think shift work affects the sleep and mental health of your employees? What sleep and mental health challenges do you think shift work might pose for shift workers as individuals? Are you aware of any shift-work related sleep and/or mental health problems within your organisation?

The impact of shift work on the organisation

- What impact, if any, do you think employee sleep and mental health related problems have on your organisation? For example, in terms of health and safety, productivity, sick leave etc.,

2) To identify potential ways to address priority areas

Current organisational efforts and barriers

- What, if anything, does your organisation currently do to help manage the impact of shift work on employee sleep and mental health? This could be something to help with sleep and/or mental health directly, or something that is done to help manage associated outcomes, such as sleepiness and fatigue, alertness, health and safety etc.,
    - You mentioned X – how successful has implementing this been?
    - Did you come across any challenges or barriers when it came to implementing X? For instance, associated costs, employee engagement etc.,
    - Was there anything you would change about the way you ran/run X to help improve the format and delivery?

Potential additional organisational efforts and barriers

- Is there anything more, or anything else, you think your organisation could (or could not) do to help manage the effects of shift work on sleep and/or mental health problems? Don't worry if you can't think of anything specific, as I will run through some potential methods in a minute to get your thoughts and insights.
    - You mentioned X, how easy do you think it would be to run something like this in your organisation?
    - What would be the challenges and barriers to implementing X in your organisation?

Gaining insights on research-backed methods and potential barriers

- Now I'm going to mention a few organisation-level methods that research suggests might help to improve the sleep and mental health of shift workers. We would like to know whether you have any thoughts or comments on these intervention elements in terms of: how easy or difficult it might be to implement them; how ready and willing your organisation would be to implement them; how much it might cost; and how well employers and employees might receive them:
    - **Making changes to shift schedules to fit with the latest research and guidelines** – for example, capping the duration of night shifts to 10hrs, ensuring any rotating shift

rosters follow a fast, forward rotation pattern; giving employees more control over their shifts in terms of timing, length and duties.
   - **Making changes to lighting** – for example, installing brighter and/or blue-enriched lighting in the workplace, providing sunglasses for the commute home.
   - **Providing sleep hygiene education and training** – for example, providing workshops and training days/handbooks as part of workplace inductions.
   - **Facilitating napping** – encouraging napping pre-night shift and providing a nap room/allowing 20–30-minute naps at specified times on night shifts.
   - **Providing access to caffeine** – ensuring employees have access to tea/coffee breaks, advising individuals to avoid consumption of caffeine towards the end of a shift.
   - **Cognitive behavioural therapy for insomnia** – for example, buying-in to an online platform and/or providing access to digital therapies for those who may be suffering from more severe sleep problems such as insomnia.
   - **Mind-body interventions** – for example, providing information and/or access to digital platforms that offer relaxation techniques such as mindfulness.
- Has this discussion prompted you to think of any additional intervention elements or methods that we could consider including in a sleep management programme? Is there anything we haven't yet mentioned that you think might be worth adding to this list or considering?
   - Is there anything we should **not** include in an intervention like this? For instance, is there anything that **wouldn't** work for your organisation and/or your employees?

<u>3) To identify potential facilitators and or modes of intervention delivery</u>
<u>4) To identify potential barriers and facilitators to implementation and engagement – what should a good sleep management programme look like?</u>

- What do you think the biggest challenges and barriers would be when attempting to implement a new sleep management programme within your organisation? For example, would it be the associated costs of making organisation-level changes, finding a suitable time and location for the delivery of certain intervention components, getting shift workers to engage with elements of the intervention etc.,
   - How ready and willing do you think your organisation would be to make such changes in the face of these barriers?
   - How ready and willing do you think your employees would be to engage in a sleep management programme like this?

Wrapping Up

- Do you have any other thoughts, comments, or suggestions that you would like to add?
