# Supplementary material for: SleepShifters: The Co-Development of a Preventative Sleep Management Programme for Shift Workers and Their Employers
Source: Int J Environ Res Public Health. 2025 Jul 25;22(8):1178. doi: 10.3390/ijerph22081178 (PMC12386607; doi:10.3390/ijerph22081178)
Supplement: Supplementary file 1 [file ijerph-22-01178-s001.zip › Supplementary File S3 – Stakeholder Reservations.pdf]

Supplementary Table:

Stakeholder Reservations Raised in Relation to Research-backed Intervention Methods; Stage 2) Understanding Stakeholder Needs, Wants & Preferences.

Research-backed Intervention Technique	Key Reservations Raised by Stakeholders
Adjusting Shift Schedules	• Employees may be required to complete reactive, emergency-based work and often work long shifts to meet the demands of client contracts.<br>• Reducing shift length or putting on additional shift rotations would require employing additional staff, which is costly in terms of recruitment, training, and pay.<br>• Many employees are reliant on the pay provided by working long-hours, nights, and/or weekends, and see reduced hours as a threat to financial security.
Controlled Light Exposure	• For those working in remote outdoor environments, implementing controlled levels of light exposure would be difficult, costly, and hazardous – especially on the railway where lighting changes could interfere with signalling and/or break regulations.<br>• Implementing brighter/blue-enriched lighting was also deemed to be costly, unnecessary, and undesirable in a factory environment, where employees were happy with current lighting levels.<br>• Employers would be willing to trial research-backed lighting changes *if* they were proven to benefit organisational outcomes *and* were financially supported by clients, regulators, researchers, or charities.<br>• The importance of keeping a dark bedroom to facilitate daytime sleeping was accepted by all employees; however, wearing sunglasses to minimise light exposure on the commute home was considered a safety risk that could increase the risk of falling asleep at the wheel.
Planned Napping	• Stakeholders responded to the notion of “planned napping” with amusement, highlighting the need to use industry-specific language when presenting ideas (i.e., “controlled rest”) and implementing initiatives and address current attitudes to sleep. Indeed, for three of the organisations, “sleeping on the job” would result in a disciplinary offense rather than sleep help and resources.<br>• Stakeholders also raised concerns about the feasibility of implementing planned napped periods, including the lack of available resting space and suitable sleeping environment; the inability to monitor and manage nap timing to ensure that employees are sleeping in line with guidance; an unwillingness to ‘pay employees to sleep’; and the potential for individuals to take advantage.
Caffeine Consumption	• Employers already provide employees with access to caffeine (i.e., kitchen facilities) and/or allow employees to bring their own drinks to work (i.e., flasks of coffee), but felt that any imposition on the timing/dosage of caffeine intake would be ill-received.<br>• Employees were open to guidance on caffeine consumption but were equally opposed to scheduled caffeine timing and/or dosage.<br>• Employees felt that current caffeine advice was conflicting (i.e., avoid caffeine several hours before sleeping, but drink caffeine on the commute home to stay alert), and welcomed clear, shift-work specific guidance.
