# Supplementary material for: SleepShifters: The Co-Development of a Preventative Sleep Management Programme for Shift Workers and Their Employers
Source: Int J Environ Res Public Health. 2025 Jul 25;22(8):1178. doi: 10.3390/ijerph22081178 (PMC12386607; doi:10.3390/ijerph22081178)
Supplement: Supplementary file 1 [file ijerph-22-01178-s001.zip › Supplementary File S4 – Questions Posed to Stakeholders at the Refinement Stage and Asssociated Feedback.pdf]

Supplementary File S4: Questions Posed to Stakeholders at the Protocol Refinement Stage and Associated Feedback

Suggested intervention components and questions put to stakeholders for further refinements; Stage 4) Stakeholder Review & Expert Refinement

Intervention Component	Questions and Discussion Prompts Posed to Stakeholders
Annual Sleep Awareness Event – consisting of an informative sleep talk, interactive exhibition, sleep-related games and giveaways.	• Who should give the talk? Who would you want to talk to if you had further questions? A famous person? Someone from a sleep charity? A sleep researcher? A wellbeing rep from work?<br>• Where should the event be held? At the workplace (the main 'hub'/head office/project site)? At an external venue?<br>• What would you like to come away from the talk knowing/having? What sleep is all about? How to improve your sleep? Sleep mask? Leaflet of sleep info? Discounts? Prizes?<br>• Do you think it should be an annual event? Mandatory attendance once? Optional/online the following year? Attendance during paid work time?
Sleep Induction Training Module for New Starters	• Do you think new starters would benefit from sleep training at the start of employment as part of their health and safety induction?<br>• Do you think new starter provisions are enough? What else could help?
Sleep Awareness Campaign – consisting of monthly themed sleep content delivered and supported through the workplace and online methods	• What do you think about having different monthly sleep topics and themes?<br>• Who would be the best person to deliver/facilitate workplace talks? A line manager? A wellbeing rep? A designated sleep rep?<br>• Do you think a 5-10min discussion could be incorporated into an onsite briefing and/or workplace meeting once a month? If not, where else?<br>• Do you think shift workers would engage in conversation prompts?<br>• Where is the best place to advertise the campaign materials?<br>• Would you share and discuss the topic with your colleagues/share posts online? Where/Would you listen to a podcast on the month's topic if it was available to you?<br>• If a topic interested you, would you follow links/go to a website to find out more?

Website – consisting of sleep resources and additional forms of support: informal (video case studies from shift working peers describing sleep experiences and practical tips) and formal (digital CBT-I platform).	• Would you go to the website if you needed help or wanted to know more about sleep?<br>• Do you think people would use the digital CBT-I platform if they needed it?<br>• Do you think employers should/would give employees the time to do CBT-I during paid worktime?<br>• Do you think the video case studies are a good idea?<br>• What other information would you like to have on a website?<br>• Would you tell your family about it and share it with them?
Sleep Scheduling App – an optional app to provide employees with tailored sleep scheduling advice and self-monitoring tools.	• Would you use an app to help you schedule your sleep around your shifts/personal life?<br>• Do you think other shift workers would be interested in this?<br>• Would you find it interesting or helpful to track your sleep/mood in this way?<br>• Do you think you would benefit from personalised sleep tips and advice?

Supplementary File S4: Questions Posed to Stakeholders at the Protocol Refinement Stage and Associated Feedback

Summary of Stakeholder Feedback & Suggested Refinements; Stage 4) Stakeholder Review & Expert Refinement

Intervention Component Category and/or Topic of Discussion	Stakeholder Suggested Refinements and Feedback
Delivery of the Sleep Awareness Event: "The Sleep Show"	• The educational talk must be given by an external sleep expert, ideally someone with experience of shift work.<br>• The event may need to be held across multiple days and/or locations to accommodate all employees.<br>• Organisations are happy to hire external venues to host health and safety-related events, e.g., hotels, football stadiums.<br>• It may be possible to 'piggy-back' and incorporate the sleep event into organisation stand-down days and/or health and safety events.
Delivery of the Monthly Themed Sleep Awareness Campaign: "Sleep Talks Campaign"	• Ensure that campaign materials do not bombard employees and/or get lost in the multitude of other ongoing campaigns.<br>• Organisations must be provided with a list of delivery methods, resources, and formats that they can 'pick and choose' from in line with their needs and preferences.<br>• A train-the-trainer handbook is needed.<br>• Charity courses (i.e., The Sleep Charity's Workplace Sleep Ambassador course) could help to train staff (e.g., line managers, current wellbeing reps) to deliver the campaign.<br>• In-person is key<br>• Podcast would be ideal to listen to on the commute.
Nutrition	• The presence of a nutritionist at the sleep awareness event would be helpful and interesting for employees.<br>• Employees could be given recipe cards and discounts on food subscriptions as part of the Sleep Toolkit.

	• Advice on food-timing and what type of food to eat and when would also be helpful – suggestion of linking it to the app.<br>• Facilitating healthy eating by increasing accessibility to healthy options in the workplace is just as important as education.
Napping Could be Reconsidered if Guidance was Provided	• Guidance on how to implement naps – or a “controlled rest” period – would be considered by employers to avoid accident risk.<br>• Employees can nap on their lunch breaks if they want to – choice.
Practical Tips Are Key	• Above anything else, employees want practical, actionable tips on what to do to improve their sleep
Use of Podcasts, Video Case Studies, and Sleep Sync App	• Some of the best received intervention elements.<br>• Gamification could help – linking the website to the app.<br>• Podcast should be conversational and have a Q&A/FAQ section so that shift workers can get answers to their questions.
Texts and Personal Items	• Text reminders and personal items may help to engage employees and act as a physical reinforcer/reminder of available resources and events – e.g., monthly text about the podcast, keyring with the sleep campaign calendar, coffee flask with sleep hygiene tips on it
Accreditation is Attractive to Employers, but not as Attractive to Employees	• Employers are incentivised by accreditations as they help to showcase their commitment to employee health and wellbeing, which makes them more competitive as a company and more attractive to new recruits.

	• Employees are wary of tokenistic accreditations and tick-box exercises – health and wellbeing awards are not surmountable to observable action and improvements in workplace culture.
Changing the Culture and Encouraging Buy-in by Getting the Language Right	• Employers (and employees) may respond better to language that is accepted and less stigmatised: health and safety, personal protective equipment, risk factor, mitigation of risk, fatigue, toolkit, safety critical.<br>• Collective ownership of employee sleep and education/involvement at all levels of the organisation will be needed to cultivate changes in workplace culture.<br>• Some individuals will not engage regardless of what you do and will dislike anything perceived as new/enforced/additional – provide the resources if they want them and avoid the use of imperative/deontic language (e.g., “rules”, “must” etc.,).
